# The accuracy of parent-reported height and weight for 6–12 year old U.S. children

**DOI:** 10.1186/s12887-018-1042-x

**Published:** 2018-02-12

**Authors:** Davene R. Wright, Karen Glanz, Trina Colburn, Shannon M. Robson, Brian E. Saelens

**Affiliations:** 1 Department of Pediatrics, University of Washington School of Medicine, M/S CW8-6, PO Box 5371, Seattle, WA 98145-5005 USA; 2 Center for Child Health, Behavior, and Development, Seattle, WA USA; 3 Department of Epidemiology and Biostatistics, Perelman School of Medicine, University of Pennsylvania, Philadelphia, PA USA; 4 Department of Biobehavioral Health Sciences, School of Nursing, University of Pennsylvania, Philadelphia, PA USA; 5 Department of Behavioral Health and Nutrition, University of Delaware, Newark, DE USA

**Keywords:** Body mass index, Body weights and measures, Misperception, Parents, Obesity, Overweight

## Abstract

**Background:**

Previous studies have examined correlations between BMI calculated using parent-reported and directly-measured child height and weight. The objective of this study was to validate correction factors for parent-reported child measurements.

**Methods:**

Concordance between parent-reported and investigator measured child height, weight, and BMI (kg/m^2^) among participants in the Neighborhood Impact on Kids Study (*n* = 616) was examined using the Lin coefficient, where a value of ±1.0 indicates perfect concordance and a value of zero denotes non-concordance. A correction model for parent-reported height, weight, and BMI based on commonly collected demographic information was developed using 75% of the sample. This model was used to estimate corrected measures for the remaining 25% of the sample and measured concordance between correct parent-reported and investigator-measured values. Accuracy of corrected values in classifying children as overweight/obese was assessed by sensitivity and specificity.

**Results:**

Concordance between parent-reported and measured height, weight and BMI was low (0.007, − 0.039, and − 0.005 respectively). Concordance in the corrected test samples improved to 0.752 for height, 0.616 for weight, and 0.227 for BMI. Sensitivity of corrected parent-reported measures for predicting overweight and obesity among children in the test sample decreased from 42.8 to 25.6% while specificity improved from 79.5 to 88.6%.

**Conclusions:**

Correction factors improved concordance for height and weight but did not improve the sensitivity of parent-reported measures for measuring child overweight and obesity. Future research should be conducted using larger and more nationally-representative samples that allow researchers to fully explore demographic variance in correction coefficients.

## Background

Measured height and weight, used in national surveillance surveys such as the National Health and Nutrition Examination Survey (NHANES) and the National Longitudinal Survey of Youth (NLSY), are used to calculate body mass index (BMI) percentile and to provide a portrait of the prevalence of childhood overweight and obesity in the U.S. [1] In-person measurement can be time- and resource-intensive. It may not always possible to obtain measured height and weight in other surveillance systems (e.g., state, county, or municipal levels) or even larger studies using remote (e.g., phone, web) data collection. Self-reported (or proxy-report such as parents reporting on their children) height and weight, have been frequently employed as substitutes for measured height and weight.

Previous studies have examined correlations between BMI calculated using parent-reported and directly-measured child height and weight and predictors of observed bias [2–9]. A review by O’Connor and Gugenheim estimated that parent-reported height and weight had sensitivity for identifying children with obesity ranging from 22 to 79% and specificity ranging from 93 to 98% [10]. While these studies each have their own strengths, they are also subject to limitations. First, many use measures of correlation such as the Pearson’s correlation coefficient or paired t-tests that fail to adequately detect levels of reproducibility [11]. Further, few studies report coefficients that can be employed to derive a correction factor for parent-reported child height and weight.

Correction factors exist for adult self-reported height and weight, but the evidence for a pediatric sample is sparse [12, 13]. The one correction factor reported for absolute child BMI (kg/m^2^) adjusts only for age; characteristics that predict variation in self-report of height and weight in adults (race/ethnicity and sex) where not included [3]. One could speculate that parent reports of child height and weight can be additionally biased by other factors such as presence of other children in the household and continued growth over time, making it even more challenging to derive a correction factor for this young population.

The present study had two objectives. First, we sought to evaluate the level of concordance between parent-reported and investigator-measured child height, weight, and derived child weight status (healthy weight, versus overweight/obese), within a large sample of 6 to 12 year olds from two metropolitan areas in the U.S. Second, if parent-reported and investigator-measured height, weight, and BMI were significantly non-concordant, we sought to develop regression models to predict corrected height, weight, and BMI estimates from parent-reported data and commonly obtained demographic factors.

## Methods

### Study population

This analysis was conducted using baseline data from the Neighborhood Impact on Kids (NIK) Study, a longitudinal observational cohort study examining associations between neighborhood characteristics and children’s weight status in Seattle/King County in Washington State and San Diego County in California. Study recruitment was conducted between 2007 and 2009. Additional details on the study, including information about the recruitment procedures, are published elsewhere [14]. The study was approved by the Seattle Children’s Institutional Review Board.

### Anthropometric measures

As part of the study eligibility process, parents were asked to report height and weight for their child during screening calls. Children below the 10th percentile BMI for age and sex based on parent-reported child height and weight were ineligible. Otherwise eligible and interested children and parents completed an in-person study visit following this phone screen. The average time between the screening call and in-person visit was 28 ± 43.9 days. The in-person visits happened in research offices or at participants’ homes based on participant preference. At the visit, the child’s height and weight was measured by trained research assistants using standard protocols [15]. Height was measured on a stadiometer (office: 235 Heightronic Digital Stadiometer; home: Portable Seca 214) and weight was measured on a digital scale (office: Detecto 750; home: Detecto DR400C). Height and weight measurements were taken three or more times until three of four consecutive measurements were within 0.5 cm or 0.1 kg of each other respectively, with the average of the measurements used.

Reported and measured height and weight were used to calculate corresponding reported or measured BMI (kg/m^2^) for parents and children. BMI percentile was calculated for children using the zanthro package in Stata (version 12) [16, 17]. Parents and children were classified as healthy weight (BMI < 25 kg/m2 or BMI percentile <85th) or overweight/obese (BMI ≥ 25 kg/m^2^ or BMI percentile ≥85th) in accordance with Centers for Disease Control and Prevention (CDC) guidelines [18, 19]. Weight and height were converted to pounds and inches for reporting purposes.

All other socio-demographic information such as parent and child age, sex, race, ethnicity, and parent education and marital status, and household income and number of children in the household was collected using a self-report survey completed by the parent following the anthropometric measurement visit.

### Analysis

Descriptive statistics (means and standard deviations for continuous variables and frequencies for categorical variables) were calculated for all study variables. Lin concordance correlation coefficients were used to assess concordance between parent-reported and measured child height, weight, and BMI [11]. A Lin coefficient of 1.0 suggests perfect concordance. In contrast to Pearson’s correlation coefficients, paired t-tests, or intraclass correlation coefficients, the Lin coefficient is designed to detect departures from a 45**°** line of absolute concordance through the origin as well as precision of the data, and is therefore a better measure of concordance and reproducibility of data than its alternatives [11]. Weight status categories (healthy vs. overweight/obese) calculated using parent-reported height and weight were compared to categories calculated using investigator-measured child height and weight to assess sensitivity and specificity of parent-reported measures.

The primary outcomes for the three regression analyses were investigator-measured height, weight, and BMI. Linear regression models were employed in analyses between the primary outcomes, corresponding parent-reported outcomes, and other covariates. Purposeful selection of covariates was used to identify variables for multivariate models using a forward selection approach. Covariates that were significantly associated with anthropometric measures in initial analyses with α ≤ 0.10 were then included in separate multivariate linear regression models for each outcome. Significance of the association between the primary outcomes and covariates in the final multivariate models was assessed at an a priori α level of 0.05.

The data were partitioned and 75% of the data were randomly selected to serve as a training data set for the development of a correction model. The remaining 25% of the data were reserved to test the accuracy of the correction model. The regression coefficients from the training data were applied to the test data set to predict corrected height, weight, and BMI values using the predict command in Stata (version 12). These predicted corrected values were then compared to the investigator-measured values to assess the accuracy of the correction model using Lin’s correlation coefficients. Accuracy of corrected BMI/BMI percentile in classifying individuals into weight status categories was assessed by calculating sensitivity and specificity.

## Results

The sample of 756 families who completed an in-person visit for the NIK study was reduced to 678 by removing cases with data that was incomplete, invalid, or produced extreme outliers in the BMI z-score calculation [17]. An additional 62 observations (8.2%) were excluded because there was a greater than two-fold ratio of parent-reported to investigator-measured height or weight. The final sample included 616 parents and children with complete data. Parents were mostly White (75%), female (86.7%), highly educated (68.3% had a Bachelor’s degree), and married (92.8%). Full sample demographic and health characteristics are presented in Table 1.

**Table 1:** Sample demographic characteristics (*n* = 616)

	Percent/Mean (SD)
Child weight status derived from measured height and weight	
Healthy Weight	74.2
Overweight	16.1
Obese	9.7
Child weight status derived from parent reports of height and weight	
Healthy Weight	71.8
Overweight	19.3
Obese	8.9
Child BMI derived from measured height and weight	17.7 (2.8)
Child BMI derived from parent reports of height and weight	17.9 (3.1)
Child BMI percentile derived from measured height and weight	61.3 (26.9)
Child BMI percentile derived from parent reports of height and weight	64.8 (26.9)
Parent BMI	
Healthy Weight	38.9
Overweight	32.6
Obese	28.5
Child age	9.0 (1.5)
Child age category	
6–9	60.7
10–12	39.3
Parent age	41.3 (5.6)
Child gender	
Female child	50.7
Male child	49.3
Parent gender	
Female parent	86.7
Male parent	13.3
Household income	
< $50,000	13.2
$50,000–$99,999	37.0
> = $100,000	49.7
Parent education	
Some college or less	31.7
College graduate	43.9
Graduate degree	24.4
Child race/ethnicity	
White Non-Hispanic	68.5
Hispanic	16.8
Other Non-Hispanic	14.7
Parent race/ethnicity	
White Non-Hispanic	75.0
Hispanic	14.2
Other Non-Hispanic	10.8
Number of children in household	
< 4	89.1
≥ 4	10.9
Parent marital status	
Married	92.8
Formerly married	4.4
Never married	1.4
Living with partner	1.4

Weight class was determined in accordance with CDC standards. For adults, healthy weight represents a BMI < 25 kg/m2, Overweight represents a BMI ≥ 25 kg/m2 and < 30 kg/m2, and Obese represents a BMI ≥ 30 kg/m2. For children, Healthy weight represents a BMI < 85th percentile for age and sex, Overweight represents a BMI ≥ 85th percentile and < 95 percentile, and Obese represents a BMI ≥ 95th percentile

**Fig. 1:**
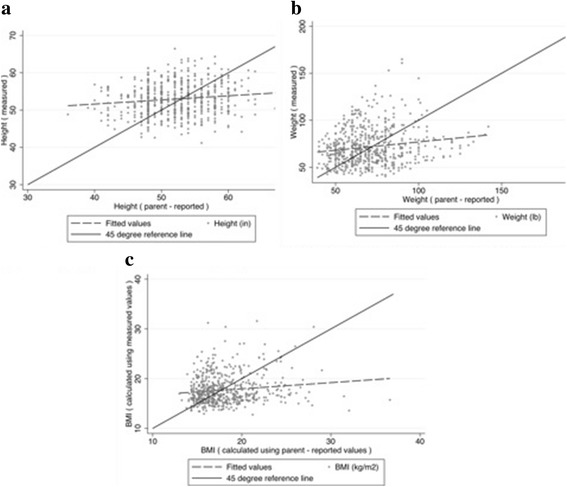
Concordance between height, weight, and BMI, calculated using parent-reported and investigator-measured height and weight. **a** Child height, ρ_c_ = 0.007 (95% CI: -0.066, 0.079). **b** Child weight: ρ_c_ = − 0.039 (95% CI: −0.113, 0.036). **c** BMI (kg/m^2^), ρ_c_ = − 0.005 (95% CI: −0.080, 0.071). NOTE: ρ_c_ is the concordance correlation coefficient, where a value close to 1.0 (and a 45° fitted line) would suggest perfect concordance

Concordance was low between parent-reported and measured height (0.007), weight (− 0.039) and BMI (− 0.005) (Fig. 1). Similar to previous findings [3], on average parents underestimated child height (− 0.82 in., 95% CI: −0.35, − 1.29); however, parents overestimated height for 6–9 year olds (1.08 in., 95% CI: 0.51, 1.65) and underestimated height for 10–12 year olds (− 3.76 in., 95% CI: −4.43, − 3.09). On average, parents underestimated child weight by 1.7 pounds (95% CI: −3.2, 0.2). In contrast to height, parents overestimated weight for 6–9 year olds (5.4 lb., 95% CI: 3.2, 7.6) and underestimated weight of 10–12 year olds (− 12.7 lb., 95% CI: −15.8, − 9.8). On average, there were no significant differences between child BMI using parent-reported versus investigator-measured height and weight (a difference of 0.24 kg/m^2^, 95% CI: −0.065, 0.55). By age group, parent report of child weight and height overestimated BMI for 6–9 year olds (0.69 kg/m^2^, 95% CI: 0.34, 1.05) and underestimated BMI for 10–12 year olds (− 0.46 kg/m^2^, 95% CI: −1.0, 0.08).

Out of 159 children classified as overweight or obese using investigator-measured height and weight, only 52 of these children were also classified as overweight or obese using parent reported child height and weight (sensitivity = 32.7%). There were 457 children classified as healthy weight using investigator-measurements and 335 were correctly classified as healthy weight based on parent-report (specificity = 73.3%). (Table 2).

**Table 2:** Sensitivity and specificity of child weight status calculated using parent-reported and investigator-measured height and weight

		Investigator-Measured		
		Overweight/Obese	Healthy Weight	Total
Parent-reported	Overweight/Obese	52	122	174
	Healthy Weight	107	335	442
	Total	159	457	616

Weight class was determined in accordance with CDC standards. Healthy weight represents a BMI < 85th percentile for age and sex, Overweight represents a BMI ≥ 85th percentile and < 95 percentile, and Obese represents a BMI ≥ 95th percentile

To improve concordance between parent-reported and measured child height and weight, the sample was parsed into training (*n* = 462) and test (*n* = 154) data sets. Linear regression models were developed on the training data set to assess which, if any, covariates were correlated with investigator-measured child anthropometrics after accounting for corresponding parent-reported values. Child gender, parent gender, the number of the children in the household, and household income were not significant univariate predictors of misreport. While many other covariates were singularly correlated with misreport, when included in multivariate models many of these covariates were found not to be independent predictors of parent misreport. For child height and weight, the corresponding parent-reported measure and child’s age were positively and significantly correlated with investigator-measured height and weight in multivariate models (R-squared = 0.62 and 0.39, respectively). BMI calculated using parent-reported child height and weight, child age, and parent education were significantly correlated with measured child BMI, with lower overall R-squared value for this model of 0.11 relative to the models for height and weight. (Table 3).

**Table 3:** Coefficients for correction model for parent-reported height and weight with 95% CIs

	Child Height Multivariate model	Child Weight Multivariate model	Child BMI Multivariate model
Intercept	29.69 [26.79, 32.60]	−4.37 [− 17.06, 8.31]	12.51 [10.00, 15.01]
Parent-reported child height/weight/BMI	0.08 [0.03, 0.12]	0.15 [0.07, 0.23]	0.08 [0.00, 0.16]^a^
Child age	2.06 [1.89, 2.22]	7.32 [6.30, 8.34]	0.43 [0.26, 0.61]
Parent age	0.02 [− 0.024,0.063]^a^	0.02 [−0.26, 0.29]^a^	0.01 [− 0.04, 0.05]^a^
Parent education			
Some college or less			ref
College			−0.61 [− 1.20, − 0.01]
Graduate degree			−1.08 [− 1.80, − 0.37]
Child race/ethnicity			
White Non-Hispanic			ref
Hispanic			0.03 [−1.02, 1.07]^a^
Other Non-Hispanic			−0.31 [−1.28, 0.65]^a^
Parent race/ethnicity			
White Non-Hispanic			ref
Hispanic			0.60 [−0.51, 1.71]^a^
Other Non-Hispanic			0.65 [−0.46, 1.76] ^a^
R^2^	0.62	0.38	0.11

The primary outcomes in these models were investigator-measured height, weight, and BMI

Child gender, parent gender, household income, and number of children in the household were considered as part of our forward selection approach, but did not make it into multivariate models

^a^ Coefficient is non-significant at α **=** 0.05 and should not be included in correction model

Coefficients derived from these regression models were applied to the covariates in the test sample to generate corrected measures of parent-reported height, weight, and BMI. For example, corrected child height was calculated as:

$$ 29.69+ 0.08\ast \left( parent- reported\ height\right)+ 2.06\ast \left( Child\  age\right) $$

Corrected measures were then compared to investigator measurements within the test sample. Mean predicted corrected parent-reported child height, weight, and BMI are reported in Table 4. While the means of the corrected values are not always closer to the investigator measured values than the original parent-reported values, concordance in the corrected test samples improved to 0.752 for height, 0.616 for weight, and 0.227 for BMI. Sensitivity and specificity of uncorrected parent-reported measures in predicting overweight and obesity among children in the test sample were 42.8 and 79.5%, respectively. Sensitivity of corrected parent-reported measures in predicting overweight and obesity among children in this test sample decreased to 25.6% while specificity increased to 88.6%.

**Table 4:** Mean parent-reported, corrected parent-reported, and investigator-measured height, weight, and BMI amongst the test sample (*n* = 154)

	Data Source		
	Parent-reported Mean (SD)	Corrected Parent-reported Mean (SD)	Investigator-measured Mean (SD)
Height (in)	53.28 (4.38)	52.76 (3.36)	52.33 (4.00)
Weight (lb)	70.62 (16.72)	70.76 (12.42)	69.99 (17.7)
BMI (kg/m^2^)	18.08 (3.08)	17.59 (0.92)	17.74 (2.89)

Inches (in), pounds (lb), kilograms (kg), meters (m), standard deviation (SF)

## Discussion

We examined concordance between parent-reported and investigator-measured child height, weight, and BMI among a sample of 6–12 year old children in two metropolitan areas in the western United States (U.S.). While sample mean values for height, weight, and BMI, and overweight/obesity prevalence estimates calculated using parent-reported and investigator-measured height and weight were similar, the sensitivity of parent-reported child height and weight for identifying overweight/obesity and concordance between parent-reported and investigator-measured height and weight on an individual child level were poor. Correction models that accounted for parent-reported measurements, child age, and parent education made significant improvements to concordance in our test sample for child height and weight, but not for child BMI. Even child BMI calculated using corrected height and weight did not result in improved sensitivity for identifying overweight or obese children, although specificity did improve.

Parents underestimated height for 10–12 years olds by 3.76 in., but only underestimated 10–12 year old child weight by 1.7 pounds. Other studies have found that parents were more likely to underestimate height than weight [3, 20]. While we hypothesized that this disparity may have been driven by confusing one child for another, the number of children in the household was not a significant predictor of misreport of child height. Children in this age group may be going through puberty and gaining height faster than they are gaining weight and parent recall may not be able to keep up with child growth trajectories. Additionally, compared to infants and younger children, routine doctor’s visits where height is routinely measured are less common for this age group, which may affect parent estimates.

A U.S. parent is more likely to report their child’s height in whole inches, meaning that if they underestimate height by an inch, they underestimate height by 2.54 cm. A parent in a country using the metric system may be able to more accurately estimate their child’s height in centimeters, a smaller unit. However, this also means that U.S. parents may be able to better estimate their child’s weight using the smaller unit of pounds compared to the larger unit of kilograms (0.45 kg per pound) compared to parents in countries using the metric system. Given these differences in measurement and potential for measurement error, study findings may be limited to the context of countries that utilize an imperial measurement system.

Given similar mean estimates of child weight, height, and BMI, from a surveillance perspective, parent-reported measurements may be adequate. However, any attempt to explore individual-level factors in relation to parent-report measures should be done cautiously given the poor individual-level concordance between parent-report and measured child anthropometrics found in this study. Dozens of national U.S. surveys including the Panel Study of Income Dynamics, the National Health Interview Survey, the Medical Expenditure Panel Survey, and the Early Childhood Longitudinal Study, to list a few, examine child development-related issues such as poverty, education, social and emotional development, and health and physical development, all of which can be mediated by or can impact obesity. Parental misreport and an inability to correct for misreport could impact our ability to understand these relationships.

In the present study, we sought to develop correction factors using commonly collected demographic information. However, some suggest that there are several reasons to avoid using correction factors, including, but not limited to heterogeneity in errors which may vary by age, race, gender, and socioeconomic status, which are readily available covariates, but also pubertal stage and exercise levels, which are harder to assess [2, 21]. Akinbambi et al. suggest that corrections are difficult to derive using linear regression even though using more complicated models may be more difficult for other investigators to use to derive corrected estimates [2]. This assertion may partially explain why we were able to improve concordance for child height and weight, but not child BMI, which is a nonlinear ratio of height and weight. Another explanation for the poor sensitivity of corrected BMI is that parents may misreport height and weight in different ways, as seen in our data when we look at misreport of height and weight by age groups. Even if we could understand the relationship between height misreport and weight misreport, it would be difficult to incorporate that information into a BMI correction factor given that BMI is a ratio that is reported as a single number.

Some caution against using correction models, but the reality is that direct measurement of child height and weight for even just a subsample of study participants can be logistically and/or fiscally prohibitive. Requiring direct measures might exclude study participants who live in rural areas, participants with inflexible schedules that would prohibit them from completing in-person assessments, and could impede studies completed via the web and on mobile devices, which offer the advantage of being able to field a survey or experiment quickly with diverse respondent samples. While direct measurements using a standardized protocol are the gold-standard for estimating obesity prevalence [22], studies with limited budgets may need to rely on other approaches.

There may be ways for investigators to improve parent-reported measurement. Concordance between parent-reported and investigator-measured height and weight may differ when the parent knows their child will be measured in-person at a later date [23], when the parent does not anticipate that their child’s measurements will be validated later [7, 10, 24], and when the parent is asked to weigh and measure their child before reporting child height and weight [4]. Therefore, suggesting to parents that measurements will be later verified or asking parents to take measurements may improve accuracy.

We may still not fully understand parents’ ability to understand numerical information or other biases that can lead to inaccurate parent report of child anthropometrics. Race [10, 23], socioeconomic status [7], and gender [7, 10] have been found to be associated with a lack of correlation between parent-reported and investigator-measured child anthropometric measurements. O’Connor and Gugenheim also found that parents overestimated their sons’ heights and underestimated their daughters’ heights, although we saw no relationship between child sex and concordance in this sample [10]. Our findings that parent education, in addition to child age, was associated with misestimation of child BMI brings to question other published correction factors that adjust height, weight, and BMI only for age [3]. There is no clear consistency between our findings and those in a study by Weden et al., which found that parents underestimate height for 2–8 year olds (− 2.1 in versus our estimate of + 1.1 in for 6–9 year olds) and 9–11 year olds (− 1.6 in versus our − 3.8 in for 10–12 year olds), overestimate weight for 2–8 year olds (+ 2.2 lbs. versus our + 5.4 lbs. for 6–9 year olds) and 9–11 year olds (+ 6.2 lbs. versus our − 12.7 lbs. for 10–12 year olds). There were fewer differences between our results and those of Weden et al. for child BMI; they estimated that parents overestimate BMI for 2–8 year olds (+ 1.5 kg/m^2^ versus our + 0.69 kg/m^2^ for 6–9 year olds) and slightly overestimate weight for 9–11 year olds (+ 0.1 kg/m^2^ versus our − 0.46 kg/m^2^ for 10–12 year olds). Some of these differences may be attributable to the fact that the Weden analysis compared two nationally-representative samples; their correction factors are population averages. While this approach has the advantage of representativeness, our concordance findings compared to our average differences suggest that population averages can inappropriately suggest a level of accuracy at the individual level that is misleading [11].

### Limitations

This analysis was subject to limitations. Height was measured by investigators in centimeters, but parents were asked to report their child’s height in inches. Therefore, investigators were able to get a more accurate height measurement than parents would have estimated. This disparity in measurement approaches could have resulted in a minor degree of child height misreport, but no more than one inch.

Secondly, the NIK sample was collected in two U.S. metropolitan areas and has limited sociodemographic diversity making it difficult to make conclusions specific to demographic characteristics such as race/ethnicity and socioeconomic status. A lack of diversity in the sample may limit the representativeness of these correction factors. An ideal correction factor would be developed using a nationally-representative sample that takes into account families of various racial/ethnic and socioeconomic backgrounds. However, we have previously observed differences in outcomes between different socioeconomic groups in the NIK sample, so the sample is not completely homogenous [25]. Lastly, data on health characteristics (e.g. age of menarche) that may impact obesity could be beneficial but are often not included in large data sets.

## Conclusions

We explored concordance between parent-reported and measured child weight and height and were able to develop a correction factor that improved the concordance between parent-reported and investigator-measured child measurements for child height and weight. However, correction factors did not improve the sensitivity of parent-reported measures for measuring child overweight and obesity. Future research should be conducted using larger and more nationally-representative samples that allow researchers to fully explore demographic variance in correction coefficients.

## Abbreviations

BMI: Body mass index
CDC: Centers for disease control and prevention
NHANES: National Health and Nutrition Examination Survey
NIK: Neighborhood impact on kids
NLSY: National Longitudinal Survey of Youth
U.S: United States

## References

[1] National Academies of Sciences E, and Medicine (2015). How modeling can inform strategies to improve population health: workshop summary.

[2] Akinbami LJ, Ogden CL (2009). Childhood overweight prevalence in the United States: the impact of parent-reported height and weight. Obesity (Silver Spring).

[3] Weden MM, Brownell PB, Rendall MS, Lau C, Fernandes M, Nazarov Z (2013). Parent-reported height and weight as sources of bias in survey estimates of childhood obesity. Am J Epidemiol.

[4] Huybrechts I, Himes JH, Ottevaere C, De Vriendt T, De Keyzer W, Cox B, Van Trimpont I, De Bacquer D, De Henauw S (2011). Validity of parent-reported weight and height of preschool children measured at home or estimated without home measurement: a validation study. BMC Pediatr.

[5] Shields M, Connor Gorber S, Janssen I, Tremblay MS (2011). Obesity estimates for children based on parent-reported versus direct measures. Health Rep.

[6] Scholtens S, Brunekreef B, Visscher TL, Smit HA, Kerkhof M, de Jongste JC, Gerritsen J, Wijga AH (2007). Reported versus measured body weight and height of 4-year-old children and the prevalence of overweight. Eur J Pub Health.

[7] Dubois L, Girad M (2007). Accuracy of maternal reports of pre-schoolers’ weights and heights as estimates of BMI values. Int J Epidemiol.

[8] Akerman A, Williams ME, Meunier J (2007). Perception versus reality: an exploration of children’s measured body mass in relation to caregivers’ estimates. J Health Psychol.

[9] Partridge RL, Abramo TJ, Haggarty KA, Hearn R, Sutton KL, An AQ, Givens TG (2009). Analysis of parental and nurse weight estimates of children in the pediatric emergency department. Pediatr Emerg Care.

[10] O’Connor DP, Gugenheim JJ (2011). Comparison of measured and parents’ reported height and weight in children and adolescents. Obesity (Silver Spring).

[11] Lin LI (1989). A concordance correlation coefficient to evaluate reproducibility. Biometrics.

[12] Jain RB (2010). Regression models to predict corrected weight, height and obesity prevalence from self-reported data: data from BRFSS 1999-2007. Int J Obes.

[13] Ezzati M, Martin H, Skjold S, Vander Hoorn S, Murray CJ (2006). Trends in national and state-level obesity in the USA after correction for self-report bias: analysis of health surveys. J R Soc Med.

[14] Saelens BE, Sallis JF, Frank LD, Couch SC, Zhou C, Colburn T, Cain KL, Chapman J, Glanz K (2012). Obesogenic neighborhood environments, child and parent obesity: the neighborhood impact on kids study. Am J Prev Med.

[15] Lohman T, Roche A, Martorell R (1988). Anthropometric standardization reference manual. Human kinetics books.

[16] StataCorp (2011). Stata statistical software: release 12.

[17] Vidmar S, Carlin J, Hesketh K (2004). Standardizing anthropometric measures in children and adolescents with new functions for egen. Stata J.

[18] About BMI for Children and Teens. http://www.cdc.gov/healthyweight/assessing/bmi/childrens_BMI/about_childrens_BMI.html. Accessed 23 Jan 2018.

[19] About BMI for Adults. http://www.cdc.gov/healthyweight/assessing/bmi/adult_bmi/. Accessed 23 Jan 2018.

[20] Gordon NP, Mellor RG (2015). Accuracy of parent-reported information for estimating prevalence of overweight and obesity in a race-ethnically diverse pediatric clinic population aged 3 to 12. BMC Pediatr.

[21] Plankey MW, Stevens J, Flegal KM, Rust PF (1997). Prediction equations do not eliminate systematic error in self-reported body mass index. Obes Res.

[22] National Academies of Sciences E, and Medicine (2016). Assessing prevalence and trends in obesity: navigating the evidence.

[23] Huybrechts I, De Bacquer D, Van Trimpont I, De Backer G, De Henauw S (2006). Validity of parentally reported weight and height for preschool-aged children in Belgium and its impact on classification into body mass index categories. Pediatrics.

[24] Garcia-Marcos L, Valverde-Molina J, Sanchez-Solis M, Soriano-Perez MJ, Baeza-Alcaraz A, Martinez-Torres A, Perez-Fernandez V, Guillen-Perez JJ (2006). Validity of parent-reported height and weight for defining obesity among asthmatic and nonasthmatic schoolchildren. Int Arch Allergy Immunol.

[25] Tandon PS, Zhou C, Sallis JF, Cain KL, Frank LD, Saelens BE (2012). Home environment relationships with children’s physical activity, sedentary time, and screen time by socioeconomic status. Int J Behav Nutr Phys Act.

